# Supplementary material for: Chemical Composition and Antibacterial Activity of Liquid and Volatile Phase of Essential Oils against Planktonic and Biofilm-Forming Cells of Pseudomonas aeruginosa
Source: Molecules. 2022 Jun 25;27(13):4096. doi: 10.3390/molecules27134096 (PMC9268626; doi:10.3390/molecules27134096)
Supplement: Supplementary file 1 [file molecules-27-04096-s001.zip › molecules-1725227-SI.pdf]

molecules

MDPI

Supplementary materials

Chemical Composition and Antibacterial Activity of Liquid and Volatile Phase of Essential Oils Against Planktonic and Biofilm-Forming Cells of *Pseudomonas aeruginosa*

Malwina Brożyna [1*], Justyna Paleczny [1], Weronika Kozłowska [2], Daria Ciecholewska-Juśko [3], Adam Parfieńczyk [4], Grzegorz Chodaczek [5], and Adam Junka [1*]

1 Department of Pharmaceutical Microbiology and Parasitology, Medical University of Wroclaw, 50-367 Wroclaw, Poland; justyna.paleczny@student.umw.edu.pl

2 Division of Pharmaceutical Biotechnology, Department of Pharmaceutical Biology and Biotechnology, Medical University of Wroclaw, 50-367 Wroclaw, Poland; weronika.kozlowska@umw.edu.pl

3 Department of Microbiology and Biotechnology, West Pomeranian University of Technology, 70-311 Szczecin, Poland; daria.ciecholewska@zut.edu.pl

4 Faculty of Medicine, Lazarski University, 02-662 Warsaw, Poland; adam.parfienczyk@lazarski.pl

5 Bioimaging Laboratory, Łukasiewicz Research Network—PORT Polish Center for Technology Development, 54-066 Wroclaw, Poland; grzegorz.chodaczek@port.org.pl

* Correspondence: malwina.brozyna@student.umw.edu.pl (M.B.); feliks.junka@gmail.com (A.J.)

A. Thyme oil

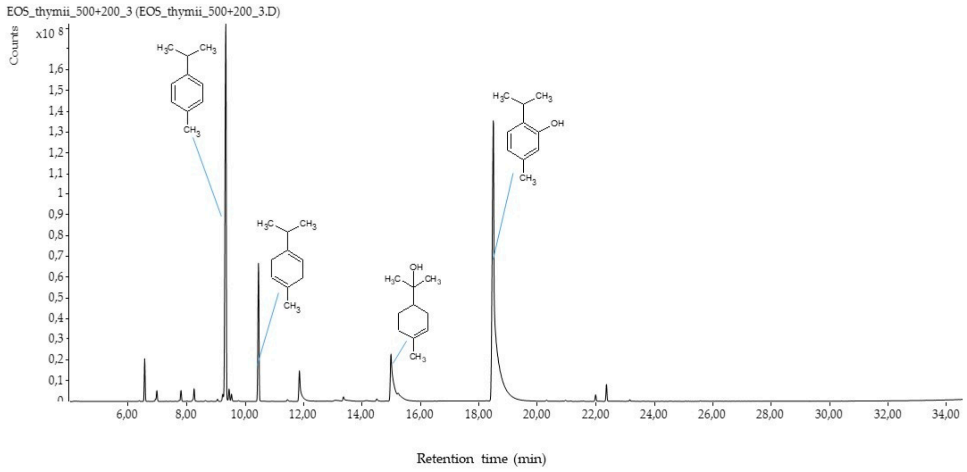

B. Tea tree oil

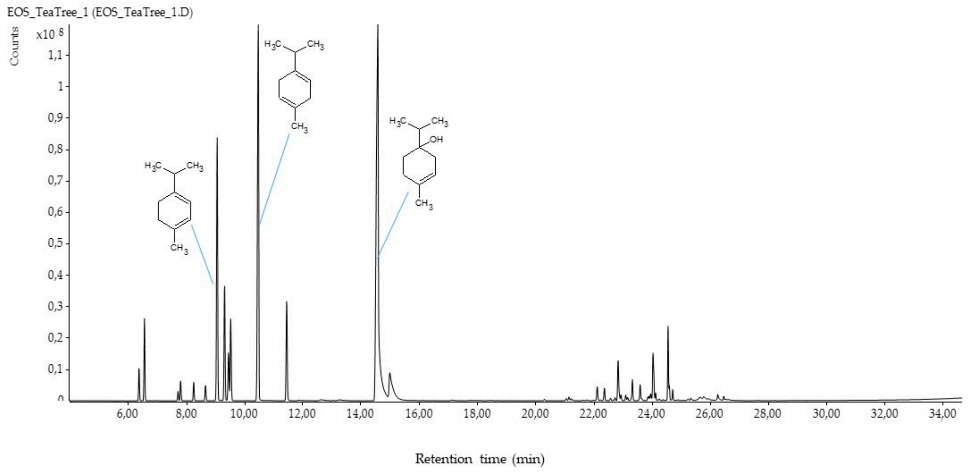

C. Basil oil

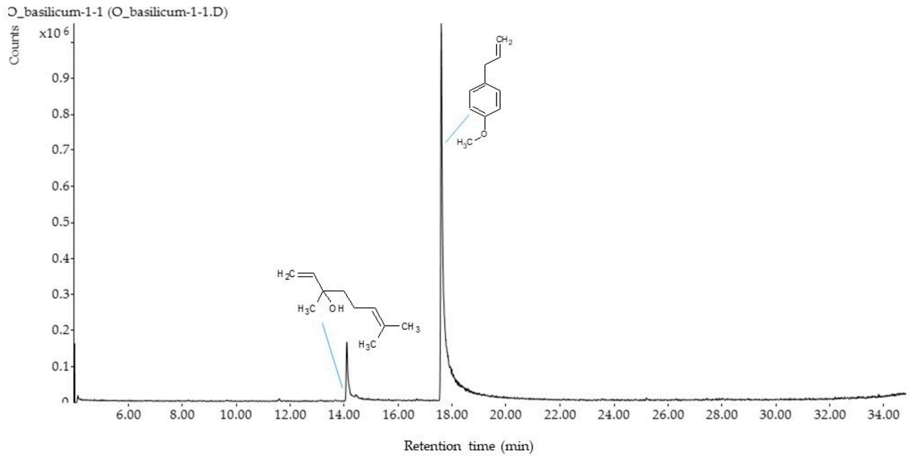

D. Rosemary oil

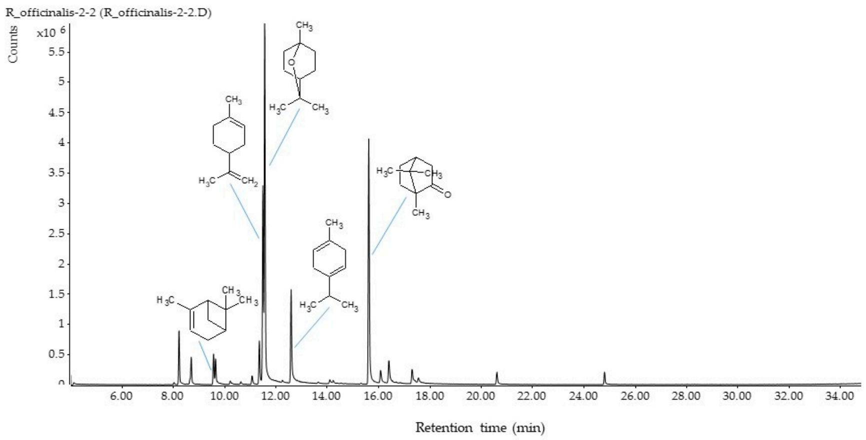

E. Eucalyptus oil

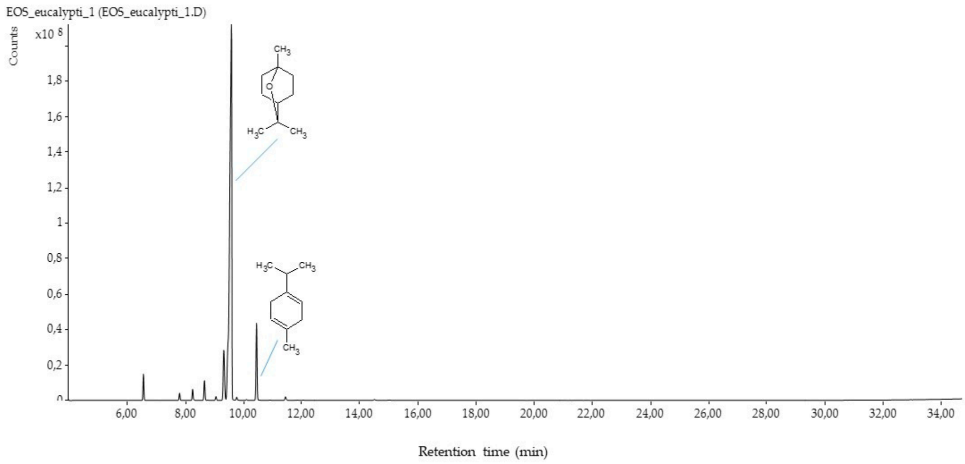

F. Menthol mint oil

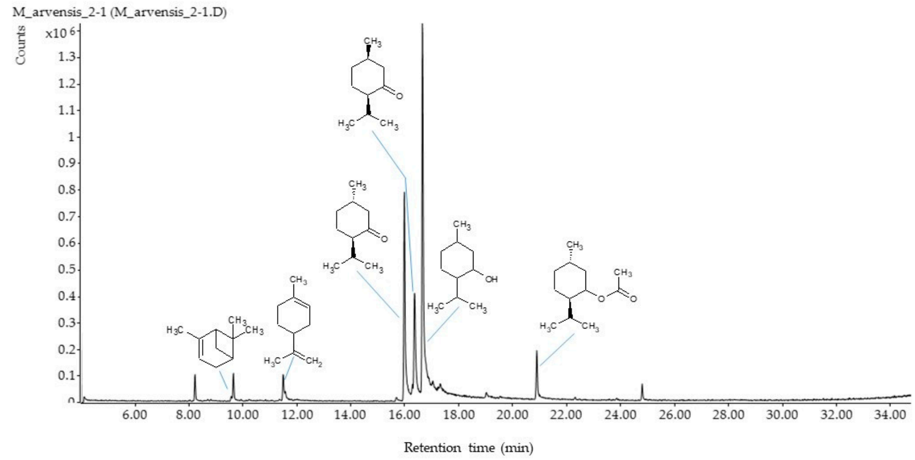

G. Lavender oil

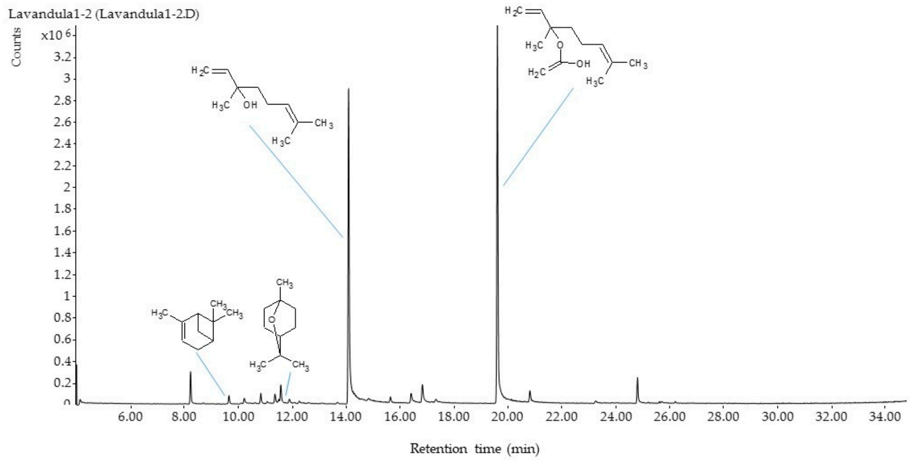

Figure S1. Chromatograms of tested EOs measured with GC-MS (Gas Chromatography-Mass Spectrometry).

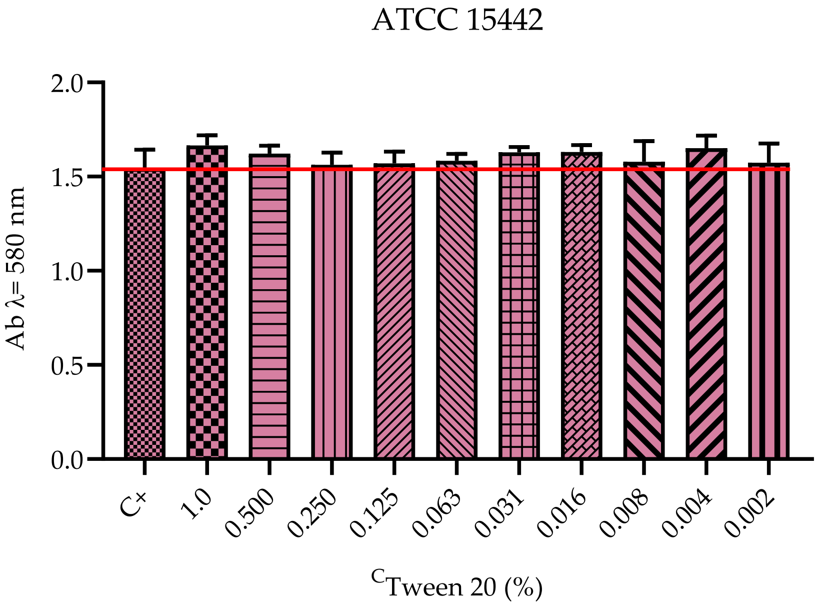

Figure S2. The antimicrobial activity of different concentrations (%) (*v/v*) of Tween 20 against planktonic forms of *P. aeruginosa* ATCC 15442 strain. Ab- absorbance after 24 h incubation, C+- untreated cells. The absorbance of untreated cells is marked with a red line.

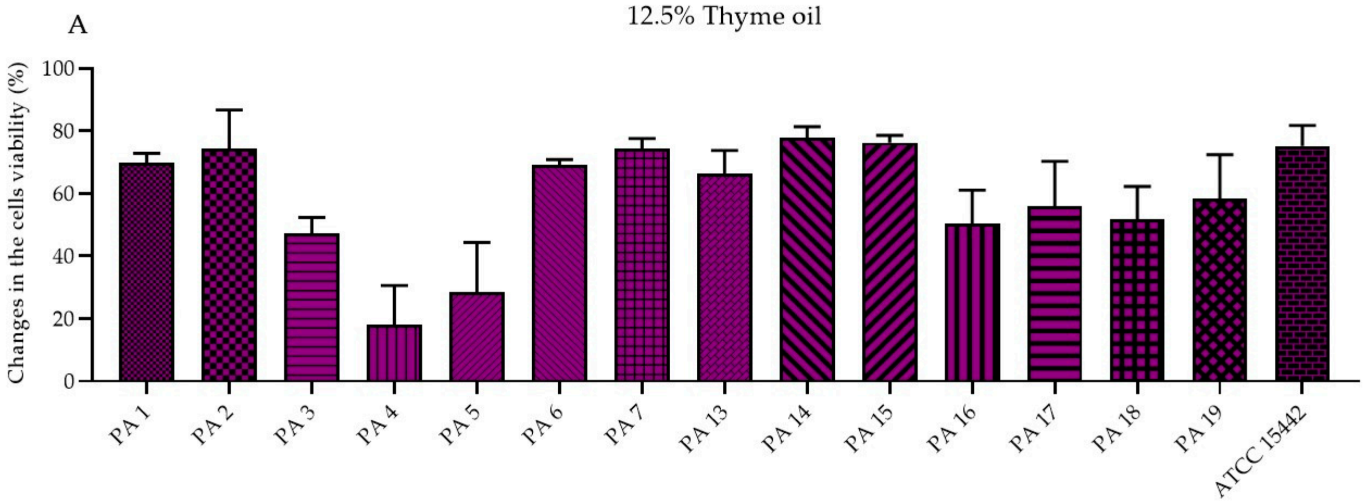

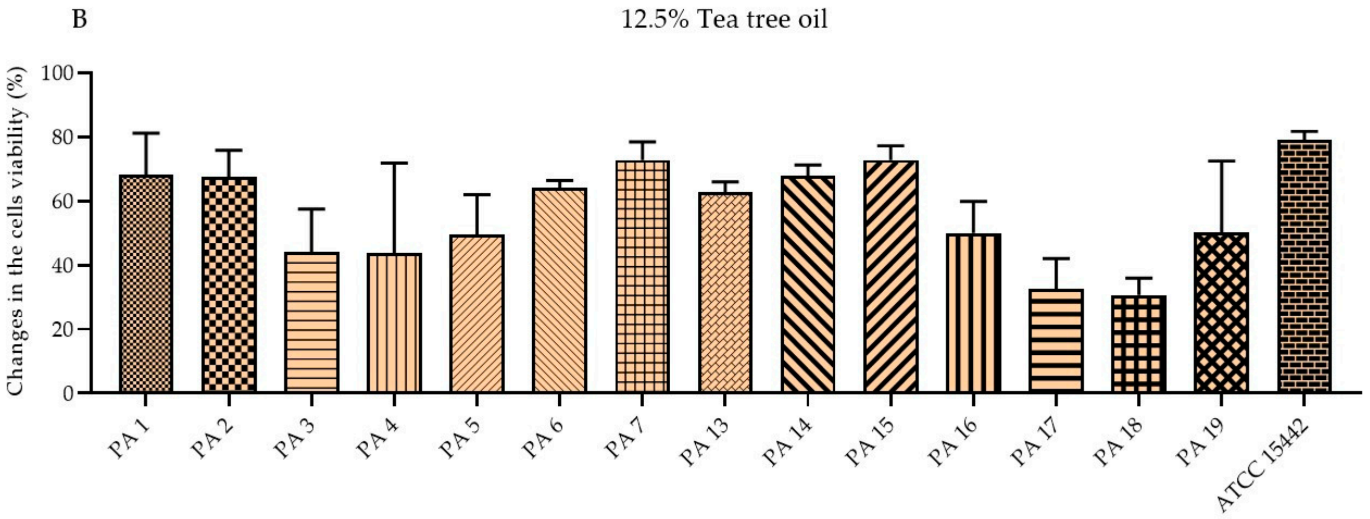
B
12.5% Tea tree oil
Changes in the cells viability (%)
100
80
60
40
20
0
PA 1
PA 2
PA 3
PA 4
PA 5
PA 6
PA 7
PA 13
PA 14
PA 15
PA 16
PA 17
PA 18
PA 19
ATCC 15442

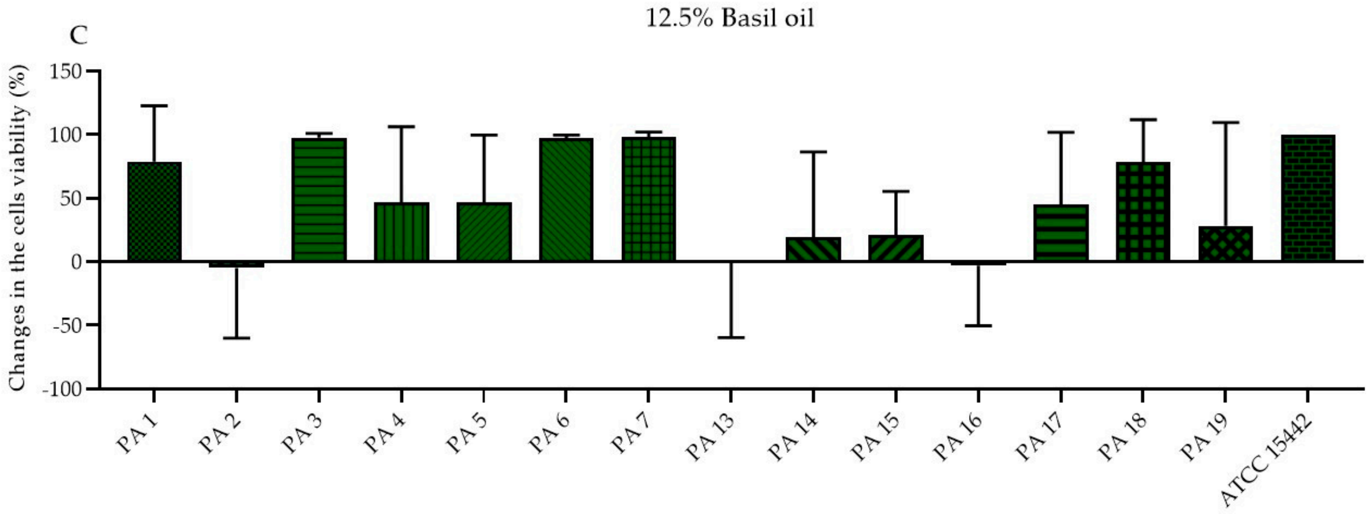
C
12.5% Basil oil
Changes in the cells viability (%)
150
100
50
0
-50
-100
PA 1
PA 2
PA 3
PA 4
PA 5
PA 6
PA 7
PA 13
PA 14
PA 15
PA 16
PA 17
PA 18
PA 19
ATCC 15442

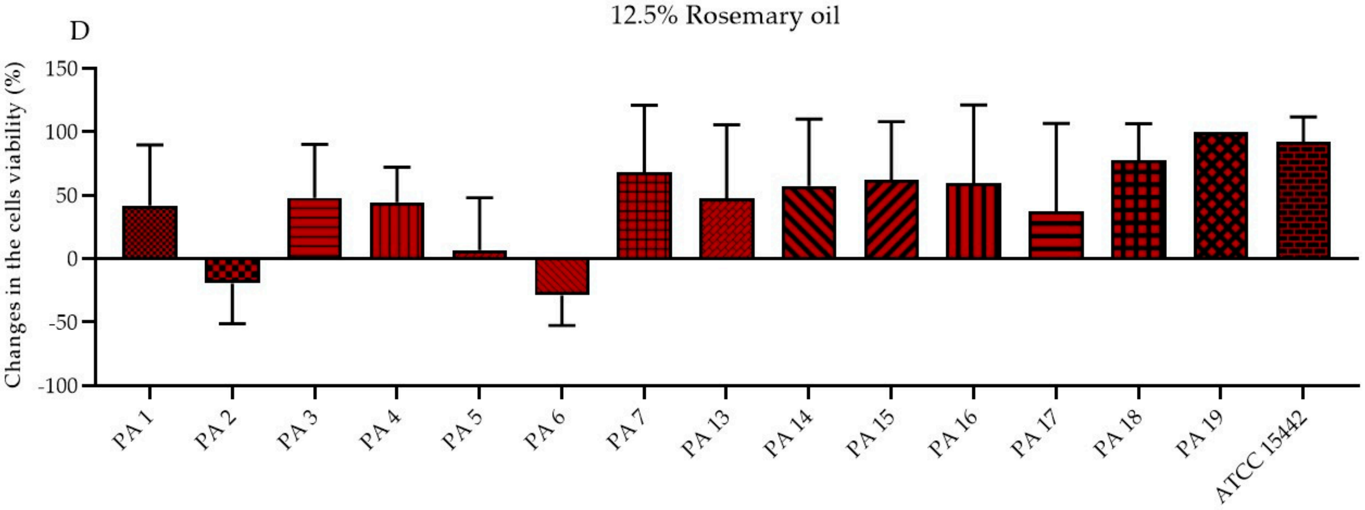
12.5% Rosemary oil
D
Changes in the cells viability (%)
150
100
50
0
-50
-100
PA 1
PA 2
PA 3
PA 4
PA 5
PA 6
PA 7
PA 13
PA 14
PA 15
PA 16
PA 17
PA 18
PA 19
ATCC 15442

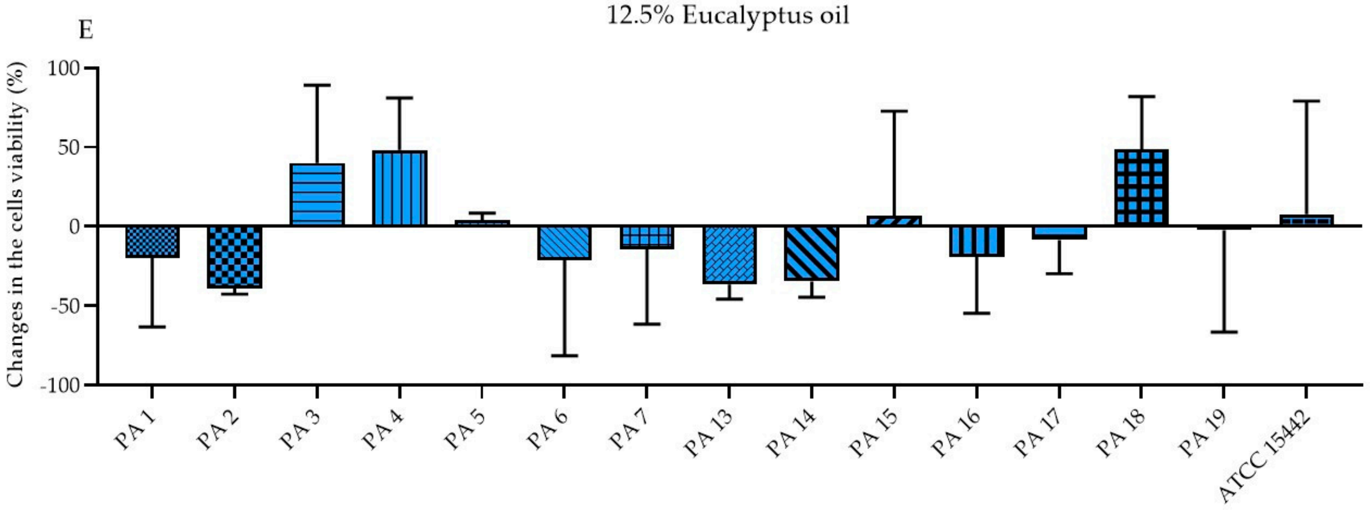
12.5% Eucalyptus oil
E
Changes in the cells viability (%)
100
50
0
-50
-100
PA 1
PA 2
PA 3
PA 4
PA 5
PA 6
PA 7
PA 13
PA 14
PA 15
PA 16
PA 17
PA 18
PA 19
ATCC 15442

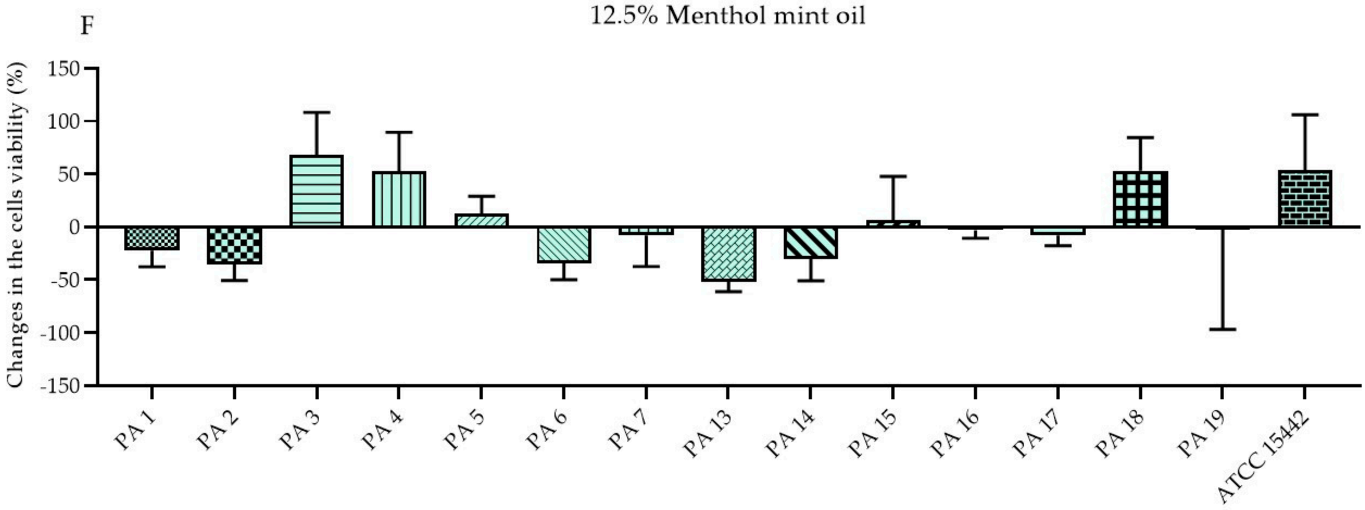

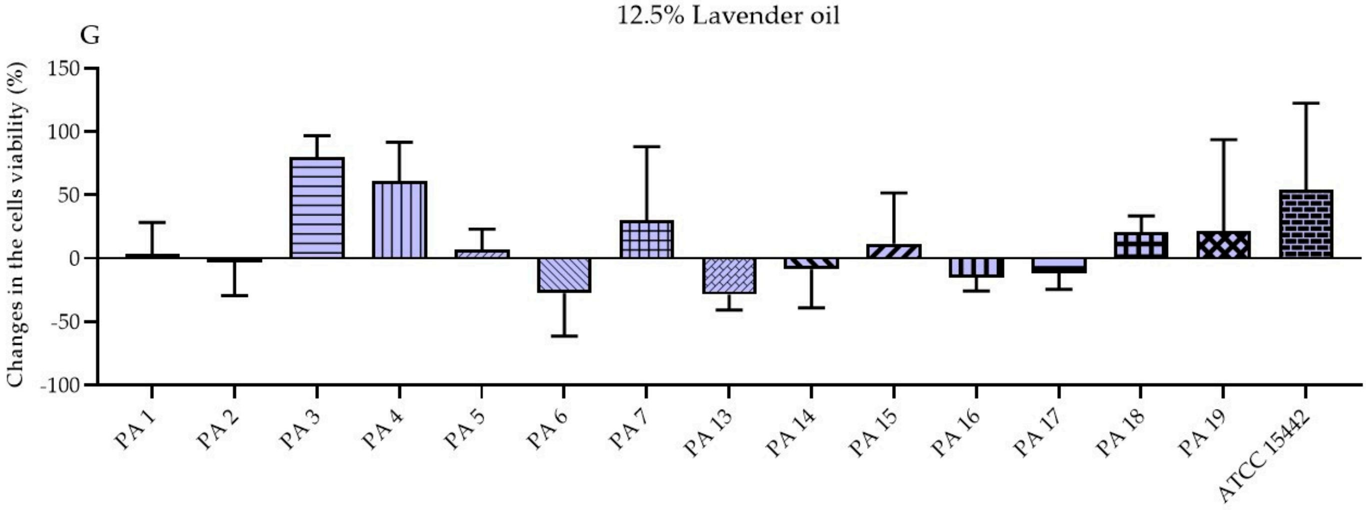

Figure S3. Changes in the biofilm-forming cells viability (%) of *P. aeruginosa* clinical (PA 1- 7, PA 13- 19) and the reference (ATCC 15442) strains after treatment with emulsified essential oils in their liquid forms in the concentration of 12.5% (*v/v*). Results of microdilution methodology with (**A**-**B**) TTC and (**C**-**G**) resazurin staining. Standard deviations are marked. The negative values indicate an increase of biofilm-forming cells' viability after their treatment with EOs in comparison to the growth control (untreated cells).

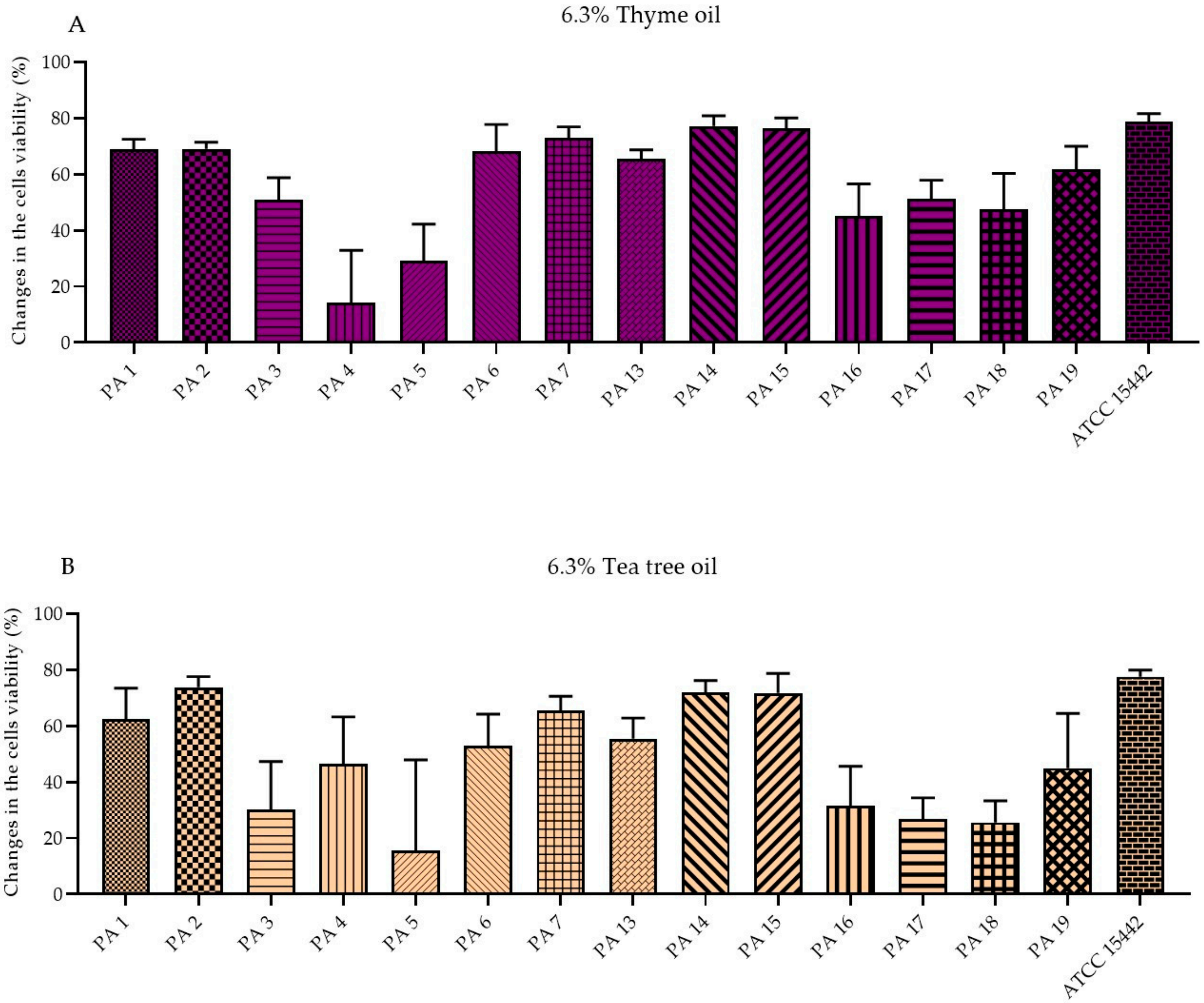
A
6.3% Thyme oil
Changes in the cells viability (%)
100
80
60
40
20
0
PA 1
PA 2
PA 3
PA 4
PA 5
PA 6
PA 7
PA 13
PA 14
PA 15
PA 16
PA 17
PA 18
PA 19
ATCC 15442
B
6.3% Tea tree oil
Changes in the cells viability (%)
100
80
60
40
20
0
PA 1
PA 2
PA 3
PA 4
PA 5
PA 6
PA 7
PA 13
PA 14
PA 15
PA 16
PA 17
PA 18
PA 19
ATCC 15442

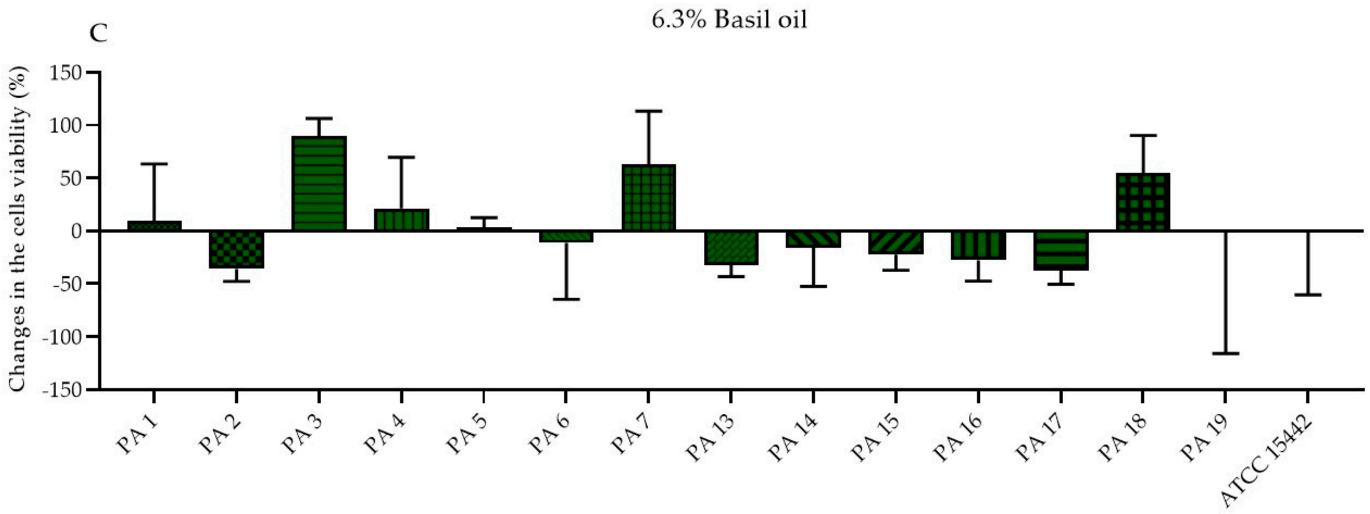
C
6.3% Basil oil
Changes in the cells viability (%)
150
100
50
0
-50
-100
-150
PA 1
PA 2
PA 3
PA 4
PA 5
PA 6
PA 7
PA 13
PA 14
PA 15
PA 16
PA 17
PA 18
PA 19
ATCC 15442

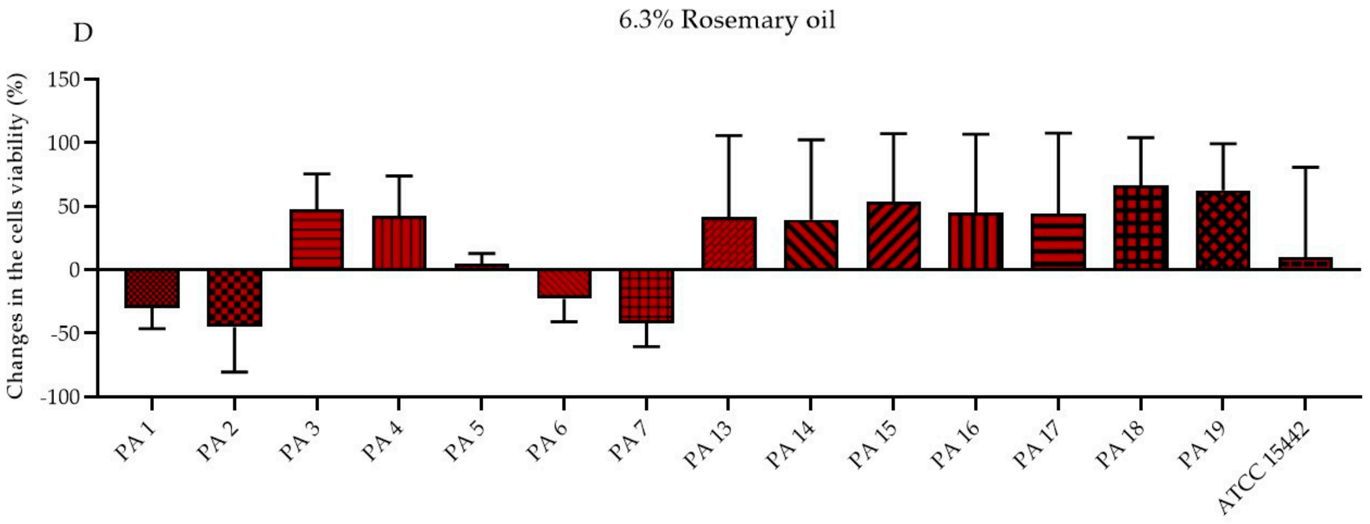
D
6.3% Rosemary oil
Changes in the cells viability (%)
150
100
50
0
-50
-100
PA 1
PA 2
PA 3
PA 4
PA 5
PA 6
PA 7
PA 13
PA 14
PA 15
PA 16
PA 17
PA 18
PA 19
ATCC 15442

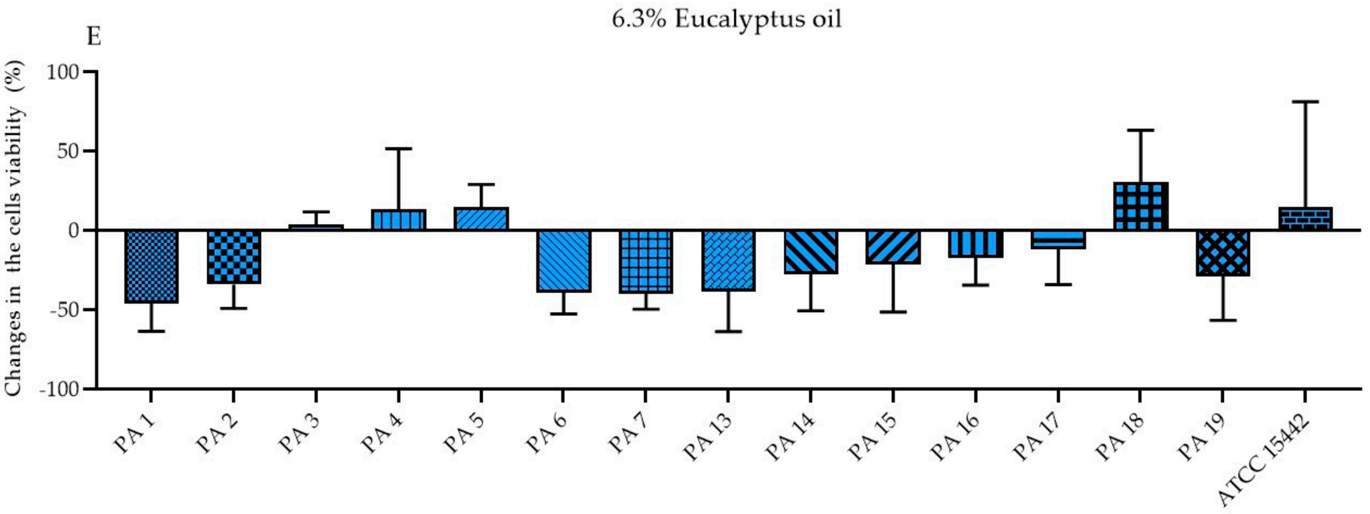
E
6.3% Eucalyptus oil
Changes in the cells viability (%)
100
50
0
-50
-100
PA 1
PA 2
PA 3
PA 4
PA 5
PA 6
PA 7
PA 13
PA 14
PA 15
PA 16
PA 17
PA 18
PA 19
ATCC 15442

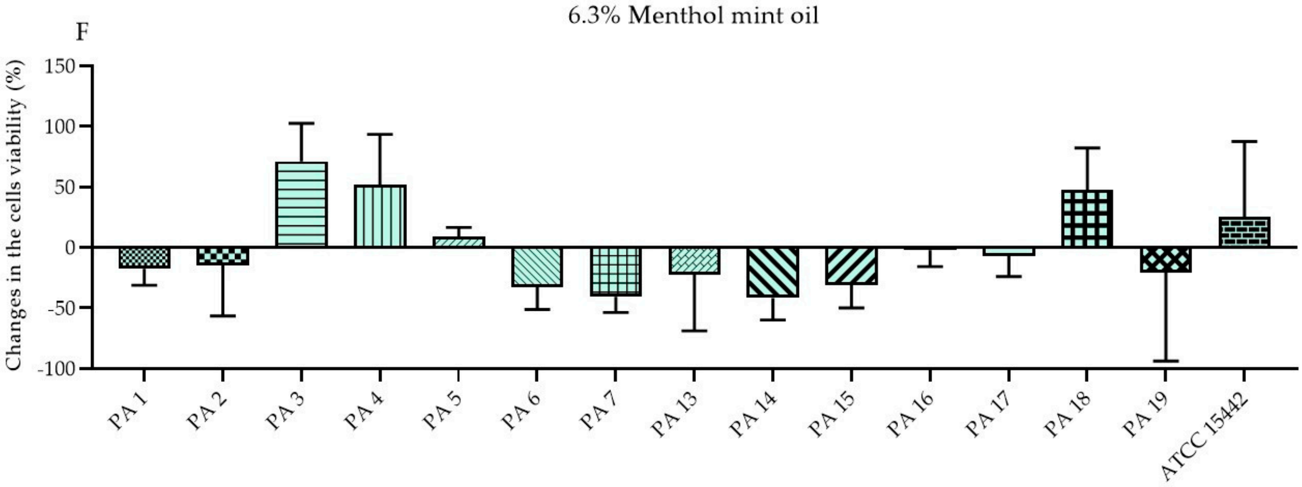

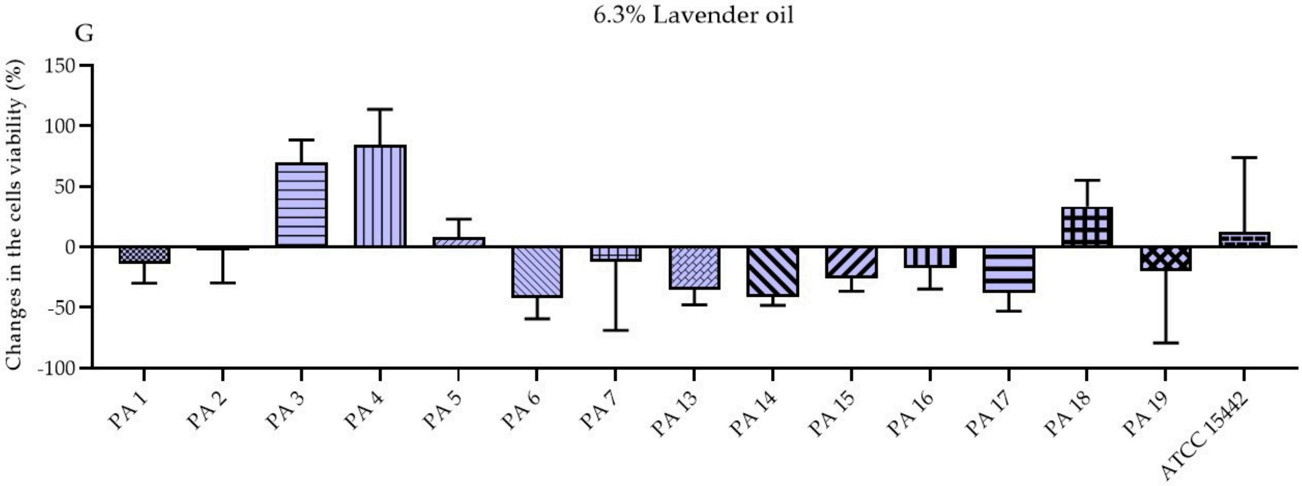

Figure S4. Changes in the biofilm-forming cells viability (%) of *P. aeruginosa* clinical (PA 1- 7, PA 13- 19) and the reference (ATCC 15442) strains after treatment with emulsified essential oils in their liquid forms in the concentration of 6.3% (*v/v*). Results of microdilution methodology with (**A**-**B**) TTC and (**C**-**G**) resazurin staining. Standard deviations are marked. The negative values indicate an increase of biofilm-forming cells viability after their treatment with EOs in comparison to the growth control (untreated cells).
